# Fixation classification: how to merge and select fixation candidates

**DOI:** 10.3758/s13428-021-01723-1

**Published:** 2022-01-12

**Authors:** Ignace T. C. Hooge, Diederick C. Niehorster, Marcus Nyström, Richard Andersson, Roy S. Hessels

**Affiliations:** 1 Experimental Psychology, Helmholtz Institute, Utrecht University, Utrecht, The Netherlands; 2 Lund University Humanities Lab and Department of Psychology, Lund University, Lund, Sweden; 3 Lund University Humanities Lab, Lund University, Lund, Sweden; 4 Tobii Pro AB, Danderyd, Sweden

**Keywords:** Eye tracking, Fixation classification, Selection rules, Minimal fixation duration, Minimal saccade amplitude

## Abstract

Eye trackers are applied in many research fields (e.g., cognitive science, medicine, marketing research). To give meaning to the eye-tracking data, researchers have a broad choice of classification methods to extract various behaviors (e.g., saccade, blink, fixation) from the gaze signal. There is extensive literature about the different classification algorithms. Surprisingly, not much is known about the effect of fixation and saccade selection rules that are usually (implicitly) applied. We want to answer the following question: What is the impact of the selection-rule parameters (minimal saccade amplitude and minimal fixation duration) on the distribution of fixation durations? To answer this question, we used eye-tracking data with high and low quality and seven different classification algorithms. We conclude that selection rules play an important role in merging and selecting fixation candidates. For eye-tracking data with good-to-moderate precision (RMSD < 0.5^∘^), the classification algorithm of choice does not matter too much as long as it is sensitive enough and is followed by a rule that selects saccades with amplitudes larger than 1.0^∘^ and a rule that selects fixations with duration longer than 60 ms. Because of the importance of selection, researchers should always report whether they performed selection and the values of their parameters.

## Introduction

Eye trackers allow researchers to study various aspects of human visual behavior and have been applied in many different settings. Eye trackers do not automatically provide the user with meaningful behaviors (e.g., fixations, saccades, blinks). The process to extract fixations from the eye-tracker signal can be conducted in different ways, which we illustrate with two example studies. In the first example, Hooge et al., (2007) were interested in how fixation durations are controlled during visual search. In their study conducted with the SMI EyeLink I, they presented the participants with visual search displays consisting of many different elements among which was the target. They were mainly interested in the fixation duration as a function of the difficulty of the foveal inspection task of the currently fixated and previously fixated search elements. To enable researchers to compute variables such as the mean fixation duration or the mean saccade amplitude, behaviors have to be extracted from the eye-tracker signal. The process used for this usually consists of two steps. In the first step, the candidates are extracted with a classification algorithm. In a second step, rules are applied to select or combine the candidates to be analyzed. Hooge et al., (2007) used a classifier based on an adaptive velocity threshold method (van der Steen and Bruno, 1995) to select saccade candidates in the eye-tracking data. In a second step, small saccades (amplitude < 1.5^∘^ and duration < 12 ms) were removed from the analysis. They operationalized fixation duration as the inter-saccadic interval. Näsänen et al., (2001) did the same and describe it as: “Samples that did not belong to a saccade were interpreted to belong to a fixation”. When a saccade was removed, the durations of the removed saccade, and of the preceding and following fixations, were summed. In contrast to others (e.g., Zani et al.,, 2020), Hooge et al., (2007) did not apply a minimal fixation duration rule. Ultimately, Hooge et al., (2007) classified and selected saccades to construct the fixations that they considered meaningful for their analysis. This meant rejecting all saccades with small amplitudes and short durations.

**Fig. 1:**
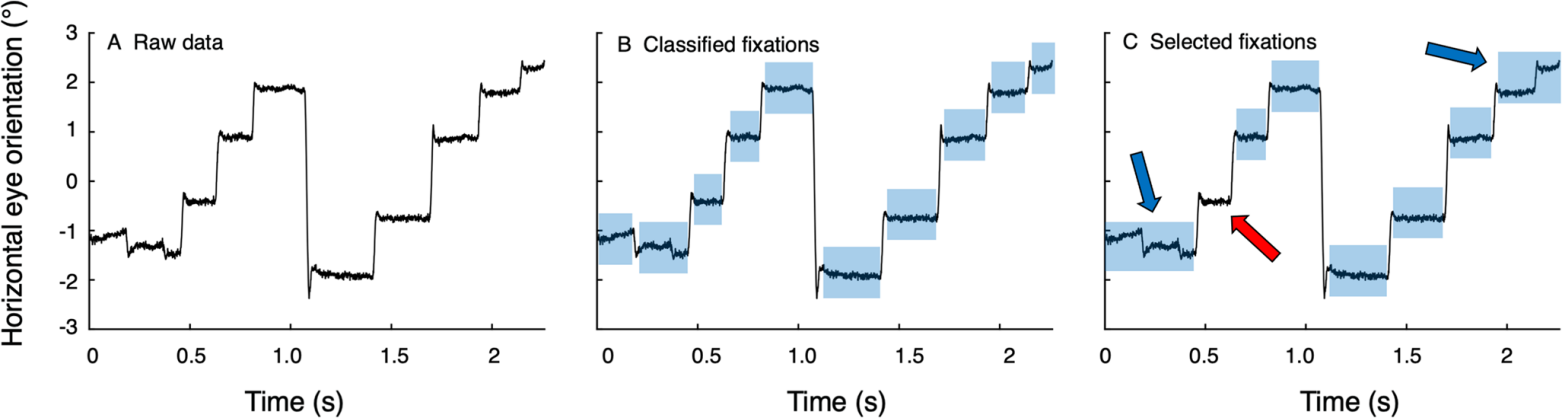
**Fixation classification flowchart**. Example flowchart showing the steps to classify fixations from a gaze position signal. A: Gaze position signal to be classified. B: Fixation candidates from the I2MC algorithm (Hessels et al., 2016b) are shaded *blue*. C: Final set of explicitly classified fixations after applying a selection rule that merged (*blue arrows*) all fixations closer than 30 ms in time and 0.8^∘^ in space. Fixations shorter than 160 ms were furthermore removed (*red arrow*). These parameters (30 ms, 0.8^∘^ and 160 ms) were chosen for illustrative purposes

In the second example, Hessels et al., (2016a) investigated the characteristics of infant saccadic search. The characteristics include fixation duration, saccade amplitude, and direction. Hessels et al., (2016a) used a Tobii TX300 and were confronted with a more difficult data processing problem than Hooge et al., (2007). The quality of infant eye-tracking data may be much lower than data recorded from adults, and this is usually reflected in a higher proportion of data loss and lower precision (for an explanation of precision, see Holmqvist et al.,, 2012; Niehorster et al.,, 2020). The precision of infant data may be so low that small saccades may be hidden in the noise (see figure 8 of Hessels et al.,, 2016b). Hessels et al., (2016a) used the I2MC fixation classifier (Hessels et al., 2016b), an algorithm that is designed to work with eye-tracking data of low quality, to find fixation candidates. Successive fixations were merged if the inter-fixation distance was smaller than 0.7^∘^ and the inter-fixation duration was shorter than 30 ms. Classified fixations were excluded if these had durations shorter than 40 ms. Hessels et al., (2016b) operationalized the saccade as the inter-fixation interval. Figure 1 shows a similar classification and selection as in Hessels et al., (2016a) but with other selection parameters.

These two studies show how different aspects of human visual behavior (control of fixation duration and characteristics of infant saccadic search) can be studied using eye trackers. In both studies, the researchers used different classification algorithms and different selection rules and parameters to determine fixations and saccades. In Hooge et al., (2007), saccade classification and selection were used to operationalize fixations indirectly. In Hessels et al., (2016a), fixation classification and selection were used to operationalize saccades indirectly. Karn (2000) describes the classifier in Hooge et al., (2007) as a saccade picker and the one used in Hessels et al., (2016a) as a fixation picker. Researchers may choose their classification algorithms based on arguments such as availability (SMI users may be using the BeGaze data processing and analysis program that came with their eye tracker), a specific quality (I2MC for infant data) or the wish to have full control over data processing and analysis by using (adapted) versions of published algorithms (e.g., the use of the NH2010 algorithm in Niehorster et al.,, 2015).

While there is a lot of interest in the literature for classification algorithms (see, e.g., Hein and Zangemeister, 2017, for an overview of approaches) less attention is given to the selection rules and their parameters, with these selection rules often being tacked onto a classifier without significant discussion or an exploration of their parameter space. Furthermore, we have previously found that the output of various classification methods (consisting of classification and selection) shows marked differences in terms of the number of classified fixations and saccades (Hessels et al., 2016b; Andersson et al., 2017). We wonder whether these differences in output are due to the different nature of the classification algorithms, or can be ascribed to the variation in the selection rule parameters that were applied. Conceptually, the selection rule parameters may have a large impact on the final eye-movement parameters such as the number of fixations, fixation duration, and saccade amplitude. For instance, a saccade-selection rule that merges fixations separated by less than 2^∘^ will remove the differences between an algorithm that classifies only saccades larger than 1.5^∘^ and another algorithm that additionally classifies saccades as small as 0.2^∘^.

### Overview of selection rules

What values for minimal saccade amplitude, saccade duration and minimal fixation duration can be found in the literature? We were surprised that many researchers do not report their selection rule parameters. We also found examples of selection rules other than the ones we already mentioned, namely maximum saccade duration (50 ms, Jacobs, 1986) and maximum fixation duration (2000 ms, Cornelissen & Vo, 2017). The following examples are not exhaustive and are meant as an illustration of the range of values. For minimal saccade amplitude (maximal inter-fixation distance), we found 0.7^∘^ (Hessels et al., 2016a), 1.0^∘^ (Kemner et al., 2008), 1.0^∘^ (Zelinsky, 1996), 2.1^∘^ (Hooge & Erkelens, 1996; 1999) and 3.0^∘^ (Diaz et al., 2013). For minimal saccade duration (maximal inter-fixation interval), we found 12 ms (Kemner et al., 2008), 22 ms (Krieber et al., 2017), 30 ms (Hessels et al., 2016a), and 50 ms (Diaz et al., 2013). For minimal fixation duration, we found 40 ms (Hessels et al., 2016a), 50 ms (Jacobs, 1986), 90 ms (Helo et al., 2014), and 230 ms (de Barbaro et al., 2011).

Various types of justifications for specific parameter choices are found in the literature. Here we will list a few. Some fixation and saccade durations (e.g., single sample saccades when recording at 500 Hz) are simply physically not possible, and thus likely reflect erroneous classification (e.g., Nyström & Holmqvist, 2010). Second, (experiment-specific) assumptions about a cognitive process may guide what saccades and fixations will be selected for further analysis. An example is the minimum fixation duration parameter in reading research (Rayner, 1999): fixations shorter than 100 ms are removed because it is thought that the decision to move on (saccade away from a word) could not have been guided by visual processing during such a short fixation. Third, only a certain subset of saccades or fixations may be relevant for the research question, e.g., saccades of specific amplitudes (see Smeets & Hooge, 2003; van der Steen & Bruno, 1995; Hooge et al.,, 2015).

### The question

In the present study, we restrict ourselves to the process of fixation determination. As shown in the examples, to determine fixations, both a fixation and a saccade classification algorithm can be used. In the latter case, a fixation is often operationalized as the inter-saccadic interval. We distinguish two cases for the first selection rule (selection of saccades with amplitudes larger than A_min_):

1. After classification of saccade candidates, the first selection rule is applied. Saccade candidates with small amplitudes (smaller than A_min_) are deleted from the list of saccade candidates. By selecting only the larger saccades, fixations (inter-saccade intervals) before and after removed saccades are merged into a longer fixation.
2. After classification of fixation candidates, the inter-fixation distance and inter-fixation duration are determined and fixations that are close to each other in time and space are combined. The inter-fixation duration is an important parameter in this process because it determines whether the inter-fixation interval qualifies as a saccade.

To limit the number of parameters in our study, we coupled the maximal inter-fixation duration (minimal saccade duration T_min_) directly to the maximal inter-fixation distance (minimal saccade amplitude A_min_) according to the following rule *T*_*min*_ = 2.2 ∗ *A*_*min*_ + 27. This formula holds for saccades with amplitudes smaller than 40^∘^ (Collewijn et al., 1988).

Since selection rules and their impact on fixation classification outcomes have not been systematically explored in the literature, it is currently not understood how classification and selection differentially contribute to how fixations are determined. In this article, we posit that the selection rule step is an important part of fixation classification that deserves more attention, both in the literature and from researchers who use fixation and saccade classifiers. The goal of this article is therefore to develop an integrated understanding of the role of selection rules in fixation classification. Such an understanding forms the basis for deciding what selection-rule parameter settings are appropriate for a given analysis. By means of experimental methods we examine the impact of selection rules on the outcomes of fixation classification. We want to answer the following question: What is the impact of the selection-rule parameters (minimal saccade amplitude and minimal fixation duration) on the outcome measure (the distribution of fixation durations) of the eye tracking study? The specific questions that we will examine are

1. What minimal saccade amplitude and what minimal fixation duration should be applied?
2. Is the impact of the selection parameters different for different classification algorithms?
3. How does the impact of selection parameters depend on quality of the eye-tracking data (precision and data loss)?

To investigate these questions, we processed and analyzed three eye-tracking data sets (consisting of data with high, lower, and low data quality) with seven classification algorithms and two selection parameters (minimal saccade amplitude and minimal fixation duration). We explored the selection parameters and we evaluated the obtained fixation duration distributions.

## Methods

### The eye-tracking data

We used three sets of eye-tracking data. The first set is the EL1000+ data set and consists of eye-tracking data of eight participants who took part in a free viewing task consisting of 104 pictures taken in the arctic area around Tromsø. Trial presentation was self-paced by the participant. Gaze of the left eye was recorded at 1000 Hz with the SR Research EyeLink 1000 Plus in desktop mode. Participants were instructed to sit as still as possible, and their heads were stabilized with a chin- and forehead rest. Distance to the screen (1920 x 1080 pixels; 53.0 x 30.0 cm) was 80 cm. The distance between the eye and the eye tracker was 55 cm. The total looking duration (of the eight participants) was 5417 s (about 90 min). To characterize the eye-tracking data quality, we computed the proportion of data loss and determined the RMS deviation (RMSD) with a moving window method (window duration 99 ms). RMSD was computed per window, then we took the median RMSD of all the windows per trial and averaged this over all 832 (= 8 x 104) trials. RMSD is 0.063^∘^ and the proportion of data loss is 0.029. We consider this eye-tracking data set the *high quality* data set.

The second set is the SMI RED250 data set and consists of eye-tracking data of the same eight participants who each conducted 104 trials. We used eye-tracking data measured from the left eye with the SMI RED250 (@250 Hz). The participant looked in her own pace at 104 other pictures of arctic scenery and was instructed to move the head a little bit and talk to the operator to make sure that the head was continuously moving with a small amplitude. Distance to the screen (1680 x 1050 pixels; 47.3 x 29.6 cm) was approximately 65 cm. The distance between the eye and the eye tracker was approximately 65 cm. The total looking duration (of the eight participants) was 8229 s (about 137 min). The window to determine the RMSD had a duration of 100 ms (25 samples). RMSD is 0.89^∘^ and the proportion of data loss is 0.074. Note that the RMSD in the position signal of the SMI RED250 set is 14.3 times higher than in the signal from the EL1000+ data set, proportion of data loss is 2.6 times higher. We consider the SMI RED250 data set the *low data quality* data set.

The third eye-tracking data set is derived from the SMI RED250 set, we refer to this set as the SMI RED250 CLEAN data set. It turned out that during many episodes the data quality was so low that all classifier algorithms (except I2MC, which was developed for classification of eye-tracking data of low quality) had difficulty dealing with these episodes (see (1) in Fig. 4). The nature of the episodes can be best described with what Abdulin et al., (2017) refer to as Rapid Irregularly Oscillating Noise of the Eye Position Signal. We decided to remove these episodes from the SMI RED250 set. To do so, we wrote a Matlab program according to the pseudocode provided by Abdulin et al., (2017). This program computes an inefficiency metric based on a moving window technique (settings: window duration = 50 ms, inefficiency threshold = 50). The detected episodes of low data quality were extended in time in both directions with 20 ms. In these episodes, gaze position coordinates were replaced by NaN (not a number). The latter increased the proportion of data loss significantly and therefore, we removed all trials (*n* = 328) having proportions of data loss exceeding 0.3. The total duration of the data set is 4277 s (about 71 min). In the SMI RED250 CLEAN data set, the RMSD is 0.29^∘^ and the proportion of data loss is 0.026. Note that the RMSD in the gaze signal of the SMI RED250 CLEAN data set is 4.7 times higher than in the EL1000+ set, the proportion of data loss is about the same.

### The classification algorithms

To determine the role of selection rules in fixation classification, we evaluated fixation duration distributions produced by seven different classification algorithms. We implemented our versions of existing and published algorithms, namely I2MC (Hessels et al., 2016b), HC2013 (Hooge and Camps, 2013), NH2010 (Nyström & Holmqvist, 2010), KF (Komogortsev et al., 2010), MST (Komogortsev et al., 2010) and CDT (Veneri et al., 2011). Here we also introduce a new algorithm called I2MW (Identification by Two Moving Windows) that is the simplest or most naive algorithm that we could think of. The method consists of two connected moving windows (e.g., 10 ms) separated by one sample. For each of the two windows, the median gaze position is computed. If the difference between the two median gaze positions exceeds a threshold (e.g., 0.1^∘^), the sample between the windows is labeled *saccade*.

**Fig. 2:**
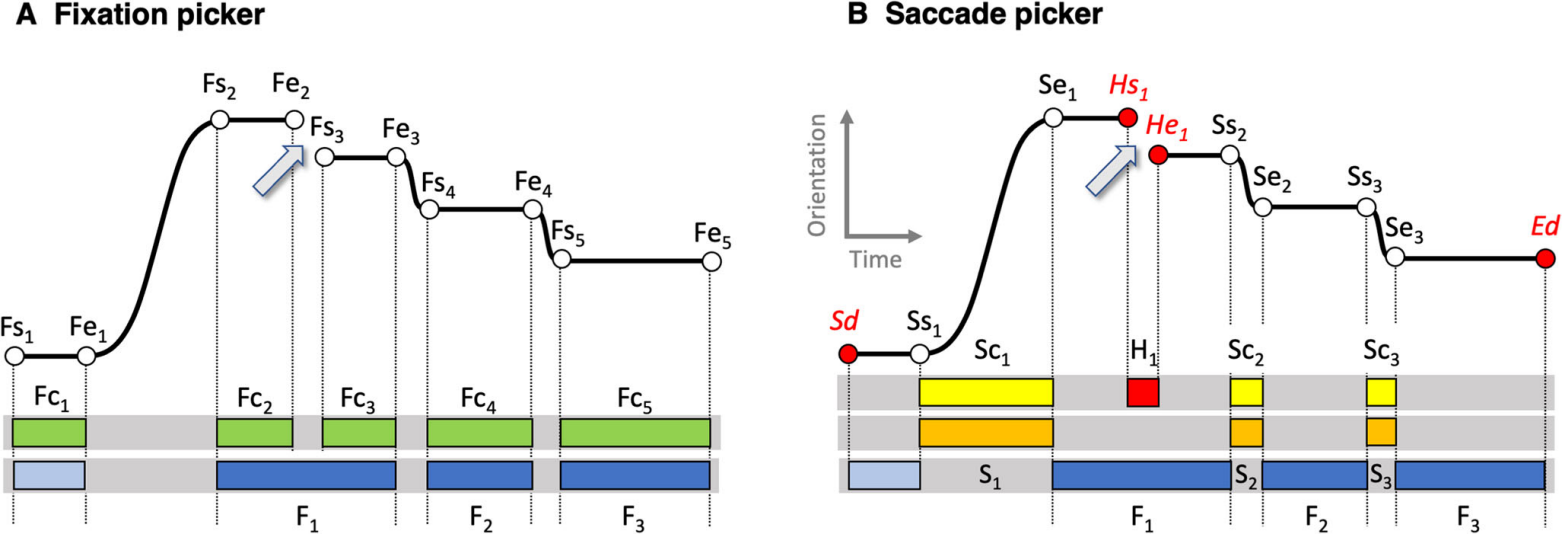
**Classifying, merging, and selecting fixations**. Example flowchart showing the steps to classify fixations from a gaze position signal. **A: Fixation picker.** The fixation picker (Karn, 2000) delivers fixation candidates (*green blocks* labeled Fc). If the inter-fixation intervals (e.g., the period from Fe_3_ to Fs_4_) exceed a minimal duration and amplitude they are not removed from the list of inter-fixation intervals. The inter-fixation interval indicated with the *gray arrow* (from Fe_2_ and Fs_3_) is removed because Fe_2_ and Fs_3_ are too close in space and time and as a result the (*green*) fixation candidates Fc_2_ and Fc_3_ are merged. From the resulting fixation candidates (*blue*) only the dark blue ones (F_1_ to F_3_) survive because they exceed the minimal fixation duration. **B: Saccade picker.** The saccade picker (Karn, 2000) delivers saccade candidates (*yellow*). Holes split fixations into two parts. To be able to remove small holes from the data, we include holes (H_1_, *red*) in the list of saccade candidates. From the saccade candidates the small or short ones are removed with selection rules that include minimal saccade amplitude and minimal saccade duration. The selected saccades (*orange*) operationalize the fixation candidates (*blue*). The minimal fixation duration rule filters out the first fixation candidate (*light blue*) and the end result of classifying saccades and selecting saccades and inter-saccade intervals are the *dark blue* fixations (F_1_ to F_3_)

Our Matlab implementations of the classifiers are available here: 10.5281/zenodo.5713693. Our implementations differ in a few ways from the originals:

1. We separated the classification and selection steps when possible. All explicit selection rules in the original algorithm were removed and replaced by our selection rules outside the algorithm. However, selection may occur implicitly. A clear example of implicit selection is that if one chooses a higher velocity threshold in a velocity threshold saccade picker, the smaller saccades are skipped. In this study, we did not investigate the selection other than by explicit selection rules.
2. Some of our seven algorithms classify both fixations and saccades. If the algorithm is used as a saccade picker we only use the classified saccade candidates, in case of a fixation picker we only use the fixation candidates. To illustrate this, we use NH2010 as a saccade picker despite that the original NH2010 classifies saccades, PSOs, fixations and noise episodes. We consider NH2010 as a saccade picker because it classifies saccades directly from the eye-tracker signal. In NH2010, the fixations are classified indirectly by choosing the periods not being saccade, PSO or noise. KF and MST directly classify both fixations and saccades from the eye-tracker signal. We choose to use them as fixation pickers. Using them as saccade pickers provided us with almost similar results.
3. Eye-tracking data may contain episodes with empty samples (NaN values for the gaze direction). Some original algorithms use interpolation to deal with holes in the data. We removed all interpolation methods. Instead, we coded the holes in the data explicitly (Fig. 2b) for saccade pickers. For fixation pickers, we applied the following rule. If a hole occurred within a fixation, we coded the start of a hole as a fixation end and the end of a hole as fixation start (Fig. 2a).
4. Some original algorithms use an explicit merge rule for fixations. We removed these. In our implementations of the algorithms, removing a hole or a small saccade by not selecting it, has the effect of merging the fixations preceding and succeeding the removed part (see Fig. 2).
5. To standardize the input for each classifier, we replaced less meaningful units (e.g., pixels and samples) by biophysically relevant units (degrees for gaze direction and minimal saccade amplitude, degrees/s for angular velocity and velocity thresholds and milliseconds for all time related parameters such as window duration for velocity filters). Parameters defined as such can be related much more easily to the values from the physiological, biological and biophysical literature. To illustrate this, one could choose 1^∘^ as the lower limit for the amplitude of voluntary saccades because this value is in the order of the span of 1.2^∘^ of the fovea (Levin et al., 2011).
6. In some original algorithms, the angular velocity is computed by a velocity filter. We chose to equalize the window duration (in ms not samples) of the filters for all algorithms and eye-tracking data sets. Since these velocity filters are implemented as symmetrical moving windows with an unequal number of samples (n + 1+n samples), we choose 21 ms (10 + 1+ 10 samples) for the velocity filters when working with the EyeLink1000+ data set and 20 ms (2 + 1+ 2 samples) for both the SMI data sets.

### Procedure

In our collection of classifiers we distinguish two different types, saccade pickers (I2MW and NH2010) and fixation pickers (CDT, HC2013, I2MC, KF and MST), for which we use different procedures (Fig. 2). If the classifier is a fixation picker (e.g., I2MC or HC2013), it provides us with fixation candidates and their starts and ends and eye orientations. In Fig. 2a, the starts and ends are indicated as Fs_2_ and Fe_2_ for the second fixation and as Fs_3_ and Fe_3_ for the third fixation. An eye tracker does not always provide valid gaze estimations, and this may result in a hole of a few empty samples in the eye-tracking data stream. Similar to small saccades, holes may divide fixations into two parts (e.g., the period between Fe_2_ and Fs_3_ in Fig. 2a). Saccade candidates are operationalized as inter-fixation intervals (this operationalization includes holes). The goal of the saccade selection rule is to remove the small saccade candidates that split fixations. To rule out that holes having long duration are included in fixations, we also apply a minimal saccade duration selection rule. The minimal duration (T_min_) of the saccade candidate is coupled to the minimal saccade amplitude (A_min_) by the formula^1^*T*_*min*_ = 2.2 ∗ *A*_*min*_ + 27. In Fig. 2a, fixation candidates 2 and 3 (green blocks with names Fc_2_ and Fc_3_) are merged into a fixation F_1_ (dark blue), because the amplitude of the saccade candidate (the inter-fixation interval indicated with a light grey arrow) was smaller than A_min_ and the duration was shorter than T_min_. The second selection rule concerns the minimal fixation duration. Figure 2a shows the selected fixations (dark blue, labelled F_1_ to F_3_).

If the classifier is a saccade picker (e.g., NH2010), it provides us with saccade candidates and their starts and ends and orientations. In Fig. 2b, these starts and ends are indicated as Ss_2_ and Se_2_ for the second saccade. Saccade pickers do not provide us with enough information to make the assumption that fixations can be operationalized as inter-saccade intervals. We added the beginning and end of the trial (Fig. 2b, Sd and Ed) and the beginnings and ends of holes in the data (Fig. 2b, e.g., Hs_1_ and He_1_). From the saccade candidates and the holes we select those that span the minimal saccade amplitude and duration by comparing their start and ending timings and orientations. In this example, three saccades candidates survive this selection, the hole does not. The selected saccades are marked orange. The fixation candidates are operationalized as the inter-saccade intervals. The second selection rule is the minimal fixation duration. Figure 2b shows the selected fixations (dark blue, labelled F_1_ to F_3_).

## Results

**Fig. 3:**
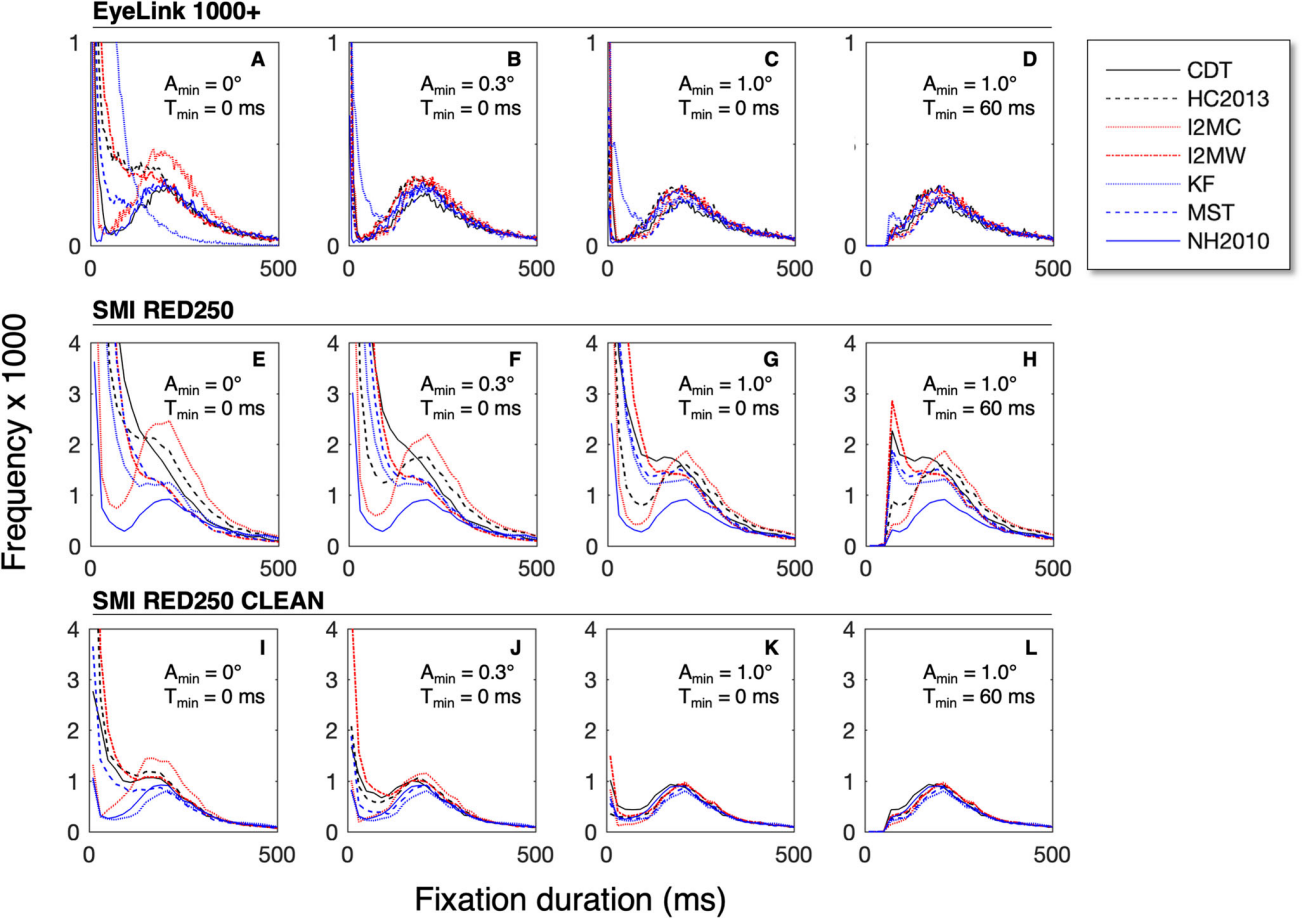
**Fixation duration distributions for different selection rule parameters**. Panels A, B, C, and D depict the distribution of fixation duration obtained from the EL1000+ eye-tracking data set for the seven classifier algorithms with different selection rule parameters. Panels E, F, G, and H depict results for the SMI RED250 data set. Panels I, J, K, and L depict results for the SMI RED250 CLEAN data set. The main conclusion of this figure is that the distribution of fixation duration is similar for different classifiers after selection rules with A_min_ = 1.0^∘^ and T_min_ = 60 ms are applied (panels D and L)

In the first analysis, we produced fixation duration distributions for each of the seven classification algorithms (Fig. 3) in the EL1000+ eye-tracking data set. We applied four different sets of selection parameters (different values for the minimum saccade amplitude A_min_ and the minimum fixation duration T_min_). Figure 3a shows the fixation duration distributions without selection (A_min_ = 0.0^∘^ and T_min_ = 0.0 ms). The different classifiers produce different fixation duration distributions. For example, the distribution of NH2010 resembles a skewed bell curve with a peak around 200 ms, while MST produces many (very) short fixations. After we removed all saccades with an amplitude below 0.3^∘^, the fixation duration distributions of the different classifiers became more similar (Fig. 3b). By removing the small saccades, many shorter fixations were merged into longer fixations (see Fig. 2). Figure 3c shows the fixation duration distributions after removal of all saccades with amplitudes smaller than 1^∘^, resulting in distributions that appear even more similar. However, it can clearly be seen that there are still many very short fixations in the fixation duration distributions (see the left part of Fig. 3c). With a T_min_ of 60 ms, all short fixations can be removed (Fig. 3d) without removing too many fixations from the bottom part of the bell shaped distribution. In summary, applying two selection rules in the order of first removing small saccades followed by removing short fixations, transforms the fixation duration distributions produced by seven different classification algorithms from very different to very similar. This can be stated in another way. In this example, the selection rules and their parameters affect the obtained fixation durations much more than the choice of the classifier algorithm.

**Fig. 4:**
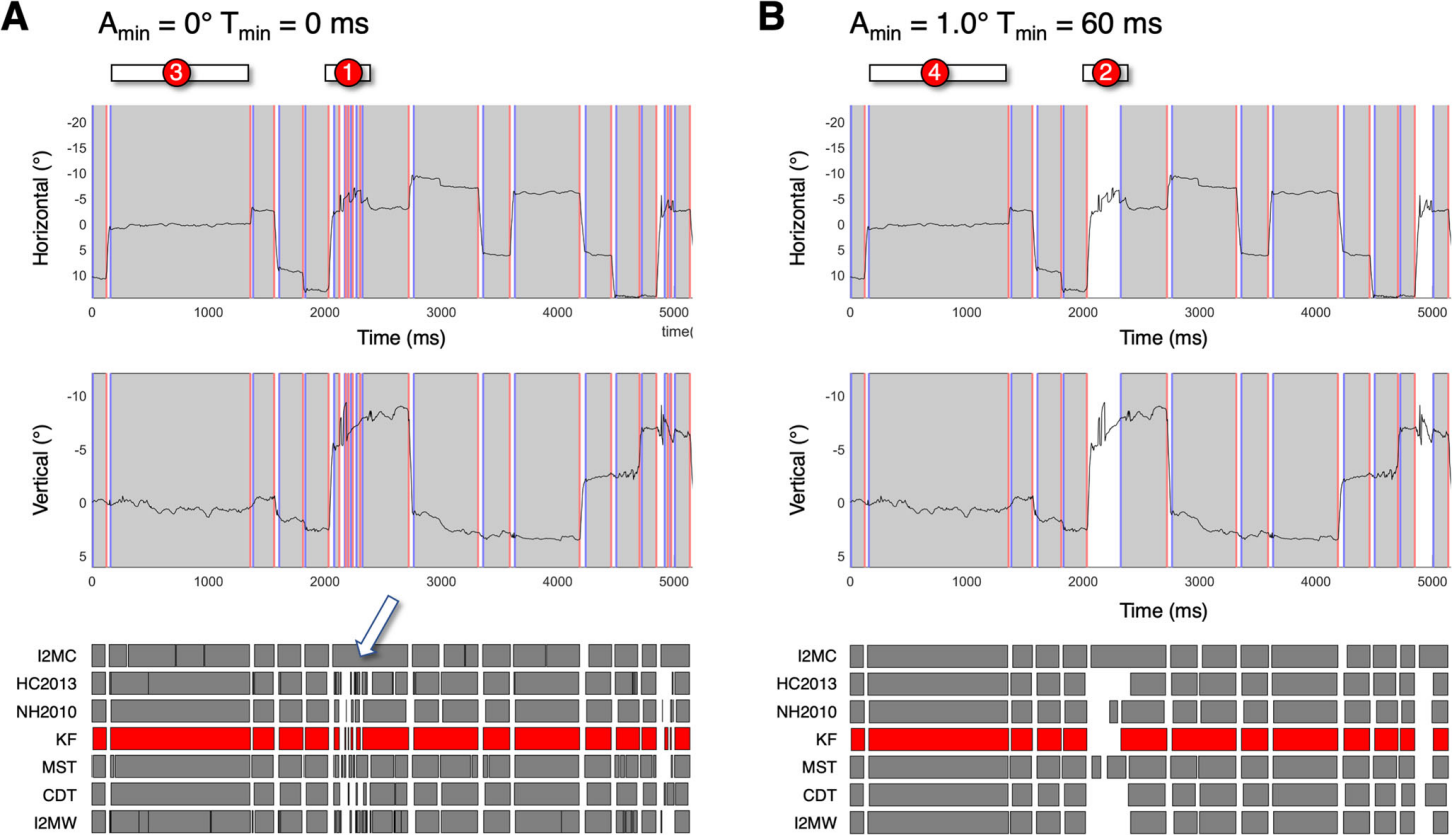
**SMI 250RED eye-tracking data set with classifications of seven algorithms.** Panel A shows horizontal and vertical components of eye orientation with fixations (*gray*) and fixation starts (*blue line*) and fixation end (*red line*). The *grey rectangles* (bottom part of panels A and B) depict the classified fixations for each of the seven different algorithms. The classified fixations of KF are marked *red*, indicating that the fixations plotted over the eye-tracking signals (top parts of panels A and B) are produced by KF. Panel A shows the unfiltered classified fixations (A_min_ = 0.0^∘^ and T_min_ = 0 ms). Panel B shows the same as in panel A but for the filtered data (A_min_ = 1.0^∘^ and T_min_ = 60 ms). The differences between the fixations between the left and the right panel are clear. Fixations in panel B are longer than these of panel A (due to merging), Panel B contains no extreme short fixations and the classified fixations of panel B are more similar between algorithms. 1) Denotes an episode of low-quality data and a clear example of rapid irregularly oscillating noise of the eye position signal (Abdulin et al., 2017). The *white arrow* points to how HC2013 classifies this episode with many very short fixations separated by fast phases with a large amplitude. This is an episode that is deleted and is not present in the SMI 250RED CLEAN eye-tracking data set. 2) Only I2MC coded the low-quality data episode as part of a longer fixation. 3) This long episode of relative stillness is classified differently by most classifiers (CDT, NH2010 and KF classify one long fixation). 4) Due to merging, the long fixation is classified similarly by all classifiers

Is the previous also true if we analyze an eye-tracking data set of lower quality? Fig. 3e, f, g and h clearly show that applying the selection rules with A_min_ = 1.0^∘^ and T_min_ = 60 ms do not result in similar fixation duration distributions for the seven classifier algorithms. Manual inspection of the gaze signals with fixation and saccade candidates plotted together, revealed that the data quality for the SMI RED250 set is too low for most of the algorithms. Except for I2MC, they produce many small fixations (see examples Fig. 4). We decided to continue with the SMI RED250 CLEAN dataset. Subsequently we also removed all trials in which the proportion of data loss exceeded 0.3. Figure 3i, j, k and l show a similar result as for the EL1000+ set. The fixation duration distributions are different when no merging (e.g., by removing small saccades) and selection are applied to the fixation candidates (Fig. 3i). After removing of the saccades with small amplitudes and removing of the fixations with short durations, the fixation duration distributions look remarkably similar (Fig. 3l).

The similarity between the fixations produced by the seven classification algorithms after selection with two rules can also be determined with an objective method. Hooge et al., (2018) developed an event-based F1-score method to compare the results of different classifiers. Table 1 contains F1 scores that quantify the agreement between classifiers for the fixation candidates obtained from the EL1000+ eye-tracking data set before merging and selection. The average F1 score is 0.47 and the F1 scores range from 0.15 (agreement between KF and CDT) to 0.77 (NH2010 and CDT). Table 2 contains F1 scores for the fixations after merging and selection with A_min_ = 1.0^∘^ and T_min_ = 60 ms. The F1 scores are much higher and range from 0.88 (CDT and KF) to 0.99 (HC2013 and I2MW) and the average value is 0.93. We repeated this procedure for the classification of the SMI RED250 CLEAN dataset. We found values ranging from 0.56 to 0.93 before merging and selection (average value is 0.69) and values ranging from 0.91 to 0.98 after merging and selection (average value is 0.94). Based on the F1 scores we conclude that the fixation duration distributions produced by seven different classification algorithms in two eye-tracking data sets are remarkably similar after merging fixations by removing saccades smaller than 1^∘^ and subsequently removing fixations shorter than 60 ms.

**Table 1:** **Event-based F1 scores for the seven classification algorithms.** The F1 scores represent agreement between classifiers for the fixation candidates obtained from the EL1000+ eye-tracking data set before merging and selection (A_min_ = 0.0^∘^ and T_min_ = 0 ms). 0.0 means disagreement, 1.0 means agreement

	I2MC	HC2013	NH2010	KF	MST	CDT	I2MW
I2MC	-	0.55	0.74	0.18	0.56	0.68	0.53
HC2013	0.55	-	0.51	0.29	0.49	0.48	0.67
NH2010	0.74	0.51	-	0.15	0.60	0.77	0.45
KF	0.18	0.29	0.15	-	0.19	0.15	0.29
MST	0.56	0.49	0.60	0.19	-	0.58	0.47
CDT	0.68	0.48	0.77	0.15	0.58	-	0.44
I2MW	0.53	0.67	0.45	0.29	0.47	0.44	-

**Table 2:** **Event-based F1 scores for the seven classification algorithms.** The F1 scores represent agreement between classifiers for the fixation candidates obtained from the EL1000+ eye-tracking data set after merging and selection (A_min_ = 1.0^∘^ and T_min_ = 60 ms). 0.0 means disagreement, 1.0 means agreement

	I2MC	HC2013	NH2010	KF	MST	CDT	I2MW
I2MC	-	0.95	0.94	0.93	0.96	0.92	0.96
HC2013	0.95	-	0.96	0.91	0.92	0.91	0.99
NH2010	0.94	0.96	-	0.90	0.93	0.90	0.96
KF	0.93	0.91	0.90	-	0.91	0.88	0.92
MST	0.96	0.92	0.93	0.91	-	0.92	0.93
CDT	0.92	0.91	0.90	0.88	0.92	-	0.91
I2MW	0.96	0.99	0.96	0.92	0.93	0.91	-

## Discussion

### Summary of results

In this study we are interested in the role of selection rules in fixation classification. We formulated three questions. 1) What minimal saccade amplitude and what minimal fixation duration should be applied? 2) Is the impact of the selection parameters different for the different classification algorithms? 3) How does the impact of selection parameters depend on quality of the eye-tracking data (precision and data loss)? The main results for this study are:

1. The fixation duration distributions produced by seven different algorithms and two selection rules with minimal saccade amplitude (A_min_ = 1.0^∘^) and minimal fixation duration (T_min_ = 60 ms) are similar (see Fig. 3 and Table 2).
2. The previous is true for eye-tracking data with high quality (RMSD = 0.06^∘^) and eye-tracking data with lower quality (RMSD = 0.3^∘^) without severe artifacts (e.g., Abdulin et al.,, 2017).
3. I2MC is the only of the seven algorithms that can deal with episodes of very low data quality (RMSD = 0.9^∘^, see Fig. 4).

### The order of application of the selection rules

In our procedure we first remove small saccades (with the effect of merging fixations) followed by removing short fixations. One may ask why we put the selection rules in this specific order. The first answer is that to our knowledge it is a convention, we have done this for decades already. We also do not know any example where the opposite order is applied. Interestingly, we find a remarkably similar approach in section 3.1.6 of the Tobii I-VT fixation filter algorithm description (Olsen, 2012) which describes a rule for discarding small saccades with two parameters (T_max_ = 75 ms; A_min_ = 0.5^∘^) followed by a rule to discard short fixations (T_min_ = 60 ms) in section 3.1.7. The EyeLink data viewer has a similar approach with merge rules followed by an option to remove short fixations (SR-Research, 2021, section 5.3.3.3).

The second answer is that removing small saccades followed by removing of short fixations leads to less data loss in the analysis. Imagine that one starts with the selection of fixations based on a minimal fixation duration. Each removal of a short fixation creates a hole in the data. Starting with the selection of saccades based on their size and duration is more obvious because in a proper operationalization of a fixation, it is included how much eye movement is tolerated within the fixation (see for an extensive discussion, cf. Hooge et al.,, 2018; Hessels et al.,, 2018). The subsequent removal of small saccades has the effect of merging fixations and does not lead to data loss. This makes even more sense if a researcher wants to keep the temporal structure of the eye tracking data intact. Examples of experimental tasks for which temporal order may be relevant are for example visual search, reading, free viewing and mind wandering.

### Is the classification correct?

Imagine one states: “The fixation duration distributions look similar, the F1 scores are very high, but perhaps all classified fixations are completely off in the same way”. In other words, the fixations may be all wrongly classified by the seven different algorithms. A solution for this problem can be a comparison with a ground truth. Before we can do that we have to agree on what may act as a ground truth. Andersson et al., (2017) used expert human coders and compared their classifications with those of ten classification algorithms. In their view, the algorithm that produced saccades and fixations closest to those coded by two human experts was the best. However, we have at least two problems with this approach:

1. A practical problem. In Hooge et al., (2018), the fastest humans coded about one fixation (e.g., fixation start or fixation end) per second. In the present study, we have 161 (= 90 + 71) min of eye-tracking data. Under the conservative assumption that our eye-tracking data sets contain about 2.5 fixations per second, this means 13.5 h of manual coding. Another problem is whether a human coder can classify this amount of data without making errors or whether one can stay unbiased (or at least maintain the same bias) (Komogortsev et al., 2010). Of course we can decrease this problem by only considering a portion of the eye-tracking data.
2. A philosophical problem. We do not acknowledge the classifications by human expert coders as the ground truth. Expert coders may have a range of ideas, definitions or operationalizations of fixations and saccades (see for extensive discussions Hooge et al.,, 2018; Hessels et al.,, 2018). Are the classifications of one expert better than the classifications of another expert? How do we know which expert provides us with “the real truth”? Or should we take a democratic approach and average the classifications of the experts? Or should we instruct the experts to classify in a certain way? But who should we then ask to instruct them?

We are not against using human coding as a pragmatic method in the evaluation and adaptation of a new classification method. Notably, the first two authors of the present study have adapted the sensitivity parameters (e.g., lambda in NH2010 and HC2013) and thresholds (e.g., CHI-squared for KF and saccade detection threshold for MST) to enable the algorithms to produce reasonable output (in their expert eyes) with two eye-tracking data sets. Manual inspection with a dedicated data viewer was part of this process.

Let us return to the original question. How do we know whether our implementations of the algorithms do provide the “correct” classification? To be honest, we do not know. Firstly, we do not know whether there is a correct classification and secondly, if the answer exists we do not know how to access it. Actually, we do not believe that there are objectively true fixations and objectively true saccades. Developers often design their algorithms with a specific goal in mind. For example, Nyström and Holmqvist (2010) designed their algorithm such that PSOs are not added to the saccade or the fixation and Engbert and Kliegl (2003) designed their algorithm to classify microsaccades.

We do not know whether our implementations of the algorithms deliver the correct classifications. What we do know is that our method of using a sensitive classifier with selection rules delivers consistent classifications under different conditions. We asked eight subjects to free view two sets of similar pictures with arctic scenery. In one set, gaze was recorded with the EyeLink 1000 plus eye tracker in the other we used the SMI RED250 eye tracker. We have no theoretical reason to believe that fixation durations should differ between these two conditions. This is exactly what our results show us. The distributions of fixations are remarkably similar between the two eye-tracking data sets. We also know that all the algorithms are already independently validated by at least their designers. There would have to be a limit to how wrong they are, and if they are wrong, they would be so consistently wrong together with the wrong intuitions of their designers that they, ironically, ultimately end up being right.

### How to design a classifier

In the present study, we showed that stripped down and slightly modified versions of seven different classification algorithms from the literature can be used to produce very similar fixation duration distributions from two different eye-tracking data sets. The trick is to use a classifier that is sensitive enough followed by selection rules that remove small and short saccades (< 1.0^∘^) and subsequently remove short fixations (< 60 ms). We showed that selection is an important process and that researchers should not worry too much about their classification algorithm as long as it is sensitive enough. Only if a researcher uses data of low quality (e.g., precision between 1^∘^ and 2.5^∘^), the I2MC classifier should be considered because the other methods cannot deal with eye-tracking data of low precision. How does a researcher know whether the current classifier is sensitive enough? Or how does a researcher know whether the quality of eye-tracking data is so good, that he should not consider using I2MC? The answer is simple: extensive manual inspection of the gaze signal with the classifications plotted on top should be a standard procedure for researchers to decide how to classify their fixations. Depending on the quality of the eye-tracking data, a researcher may also decide to use the method of Abdulin et al., (2017) to remove what they refer to as “rapid irregularly oscillating noise of the eye position signal”. In the present study, this happened to be an effective method to clean up eye-tracking data from the SMI RED250.

### Advantages of our implementations of existing algorithms

What is the difference between our implementations of the classification algorithms with selection rules at the end, versus an algorithm with all these selection rules baked-in or implicit to the algorithm? Is it only a difference in transparency? Probably, but transparency is not a goal in itself. Researchers should know their eye-tracking data, their classifiers (and settings) and should be aware of the effect of the selection rules as a function of their parameters. Researchers should have a clear idea what they expect from their classification (including selection). It does not matter whether they use closed-source, published or our implementations of existing classifiers. A deviation from the expected outcome (e.g., an unexpectedly high number of small saccades and/or short fixations should make a researcher cautious and willing to inspect the eye tracker signals with classifications plotted on top. Our implementations have at least two properties that make them preferable over the originals in our opinion: 1) they accept episodes of data loss and deal with them without interpolation and 2) they are fed with biophysically and physiologically relevant parameters that can be inspired by or found in the literature (e.g., span of the fovea, relations between amplitude, duration, and velocity of saccades). We also introduced I2MW (designed in a 10-min group discussion between the authors) that performs equally well as many of the more fancy algorithms. I2MW does not contain any “magic” and has only a few easy to adjust parameters. We recommend that researchers look at their gaze signal. Researchers should always report whether they performed selection and if they do, they should report the values of their parameters.

## Conclusions

Selection rules play an important role in merging and selecting fixation candidates. For eye-tracking data with good to moderate precision (RMSD < 0.5^∘^), the classification algorithm of choice does not matter too much as long as it is sensitive enough. Two selection rules with minimal saccade amplitude (A_min_ = 1.0^∘^) and minimal fixation duration (T_min_ = 60 ms) give remarkably good results in two representative eye-tracking data sets. Researchers should always report whether they performed selection and report what parameters they used.

## Footnotes

1 This formula is adapted from Collewijn et al., (1988), who describe the relation between saccade duration and saccade amplitude.

## Declarations

This research project does not belong to the regimen of the Dutch Act on Medical Research Involving Human Subjects, and therefore there is no need for approval of a Medical Ethics Committee. However, the present study is approved by the Ethics Committee of the Faculty of Social and Behavioural Sciences of Utrecht University and filed under number 21-0091.
